# Identification of fever and vaccine-associated gene interaction networks using ontology-based literature mining

**DOI:** 10.1186/2041-1480-3-18

**Published:** 2012-12-20

**Authors:** Junguk Hur, Arzucan Özgür, Zuoshuang Xiang, Yongqun He

**Affiliations:** 1 Department of Neurology, University of Michigan, 48109, Ann Arbor, MI, USA; 2 Department of Computer Engineering, Bogazici University, 34342, Istanbul, Turkey; 3 Unit for Laboratory Animal Medicine, University of Michigan, 48109, Ann Arbor, MI, USA

## Abstract

**Background:**

Fever is one of the most common adverse events of vaccines. The detailed mechanisms of fever and vaccine-associated gene interaction networks are not fully understood. In the present study, we employed a genome-wide, Centrality and Ontology-based Network Discovery using Literature data (CONDL) approach to analyse the genes and gene interaction networks associated with fever or vaccine-related fever responses.

**Results:**

Over 170,000 fever-related articles from PubMed abstracts and titles were retrieved and analysed at the sentence level using natural language processing techniques to identify genes and vaccines (including 186 Vaccine Ontology terms) as well as their interactions. This resulted in a generic fever network consisting of 403 genes and 577 gene interactions. A vaccine-specific fever sub-network consisting of 29 genes and 28 gene interactions was extracted from articles that are related to both fever and vaccines. In addition, gene-vaccine interactions were identified. Vaccines (including 4 specific vaccine names) were found to directly interact with 26 genes. Gene set enrichment analysis was performed using the genes in the generated interaction networks. Moreover, the genes in these networks were prioritized using network centrality metrics. Making scientific discoveries and generating new hypotheses were possible by using network centrality and gene set enrichment analyses. For example, our study found that the genes in the generic fever network were more enriched in cell death and responses to wounding, and the vaccine sub-network had more gene enrichment in leukocyte activation and phosphorylation regulation. The most central genes in the vaccine-specific fever network are predicted to be highly relevant to vaccine-induced fever, whereas genes that are central only in the generic fever network are likely to be highly relevant to generic fever responses. Interestingly, no Toll-like receptors (TLRs) were found in the gene-vaccine interaction network. Since multiple TLRs were found in the generic fever network, it is reasonable to hypothesize that vaccine-TLR interactions may play an important role in inducing fever response, which deserves a further investigation.

**Conclusions:**

This study demonstrated that ontology-based literature mining is a powerful method for analyzing gene interaction networks and generating new scientific hypotheses.

## Background

Fever, or pyrexia, is an abnormal elevation of body temperature, usually a result of a pathologic process. Normal body temperature is ranged 98-100°F (36.5-37.5°C), and temperatures above this range are usually considered febrile. Increased body temperature usually indicates possible presence of infection or sepsis. Once an infection occurs, the body responds to control the infection, often resulting in increased temperature. The fever in response to infection is likely a cure to remove infection and create a favorable environment for immune compartments such as white blood cells [1]. Nevertheless, a long-lasting fever can cause devastating effects; therefore, reducing fever either with medication or physical cooling methods remains a common practice.

Fever is induced by a substance called pyrogen, which can be either endogenous or exogenous to the body. Endogenous pyrogens are cytokines produced by phagocytic cells. Major endogenous pyrogens include interleukin-1 α/β (IL-1A/B), interleukin 6 (IL6), and tumor necrosis factor alpha (TNFA) [2]. Minor pyrogens include interleukin-8 (IL8) and interferon-α/β/γ (INF-A/B/G). These major or minor endogenous pyrogens are released into the general circulation, migrate to the circumventricular organs of the brain, and activate the arachidonic acid pathway. Exogenous pyrogens, such as lipopolysaccharide (LPS) from Gram-negative bacteria, can interact with host cell immune factors, such as LPS-binding protein (LBP), and trigger the release of endogenous factors, which in turn activate the arachidonic acid pathway [2]. The arachidonic acid pathway is mediated by phospholipase A2 (PLA2), cyclooxygenase-2 (COX-2), and prostaglandin E2 synthases (PTGES) [3]. These enzymes mediate the synthesis and release of prostaglandin E2 (PGE2). PGE2, the ultimate mediator of the fever response, stimulates the hypothalamus in the brain to generate a systemic response to increase the body temperature. The hypothalamus is responsible for coordinating complex heat effector mechanisms [4]. While the general fever pathway has been well studied, more detailed gene interaction networks associated with fever under different experimental conditions are typically unclear.

Vaccination is the process of administration of a vaccine to a host to stimulate the host immune system to develop adaptive immunity to a pathogen or against a specific disease (e.g., cancer). The immunological process after vaccination involves many immune cells including macrophages, dendritic cells, and lymphocytes. These immune cells can undergo certain levels of inflammation enhanced by various immune factors. Many vaccines can frequently cause fever [5-8]. Our main hypothesis is that vaccination stimulates inflammatory fever responses that may be required for the induction of protective immunity or act as an undesired adverse effect. However, how vaccination perturbs certain fever-related genes to cause the adverse event is still unclear. This study targets learning more about the genetic interaction processes behind the vaccine-induced fever inflammatory responses.

We previously demonstrated that high-throughput literature mining and the use of ontology can significantly enhance our understanding of vaccine research [9-11]. First, we developed a literature-based discovery (LBD) approach integrating text mining with network centrality analysis, which was successfully applied to a study investigating the role of interferon-gamma (IFN-γ) in vaccine-mediated gene-interaction networks [9]. Here an interaction network represents a network with various direct and indirect interactions. Gene-gene interaction networks were generated from the biomedical literature using natural language processing (NLP) techniques, and the most important genes in these networks were identified by network centrality analyses using four types of centrality measures: degree, eigenvector, closeness, and betweenness. Integrating these multiple centrality-based core gene sets in the vaccine subdomain resulted in the identification of a vaccine-specific sub-network of IFN-γ [9].

In an extended study, the application of the Vaccine Ontology (VO) significantly improved the analysis of the vaccine-specific IFN-γ sub-network [11]. A biomedical ontology is a controlled set of terms and relations that represent entities in the scientific world (e.g., the vaccine domain) and how they relate to each other. Therefore, a biomedical ontology can be considered as a well-defined machine-parsable “terminology” of terms together with logically defined relations between these terms. VO is a community-based ontology in the domain of vaccine and vaccination [12]. Developed in Web Ontology Language (OWL), VO provides a logic based framework for describing associations of vaccines (including licensed vaccines, vaccines in clinical trial, or vaccines proven in research), vaccine components, microbial genes engineered for vaccine development, and vaccine-induced host gene and immune responses. The relations between different VO vaccine terms have been logically defined and support advanced semantic reasoning.

Specifically, VO can be useful in two ways in the literature mining-based approach for vaccine research [11]. First, VO provides an asserted list of specific vaccines and synonyms of each vaccine, allowing the extraction of interactions between IFN-γ and specific vaccines (instead of the general term “vaccine” from sentences). Secondly, the rich semantic constructs in the VO OWL format (*e.g.*, necessary and sufficient conditions) provides logical definitions (axioms) of vaccine attributes and enables the inference of the subclasses of additional parent terms (*e.g.*, “inactivated vaccines”). VO includes a hierarchical structure based on transitive “is_a” relation. This relation indicates that a child term (*e.g.*, “*M. tuberculosis* vaccine BCG”) is always a parent term (*e.g.*, “*M. tuberculosis* vaccine”) with a specific restriction. The attributes of a specific vaccine are also defined in VO. For example, BCG is defined to have the quality of “virulent” and “viable (synonym: live)”. Necessary and sufficient conditions can also be used for inference. For instance, although BCG is not asserted as a child of “live attenuated vaccine”, the BCG “is_a” hierarchy definition combined with its attributes will allow a reasoner to infer BCG as a “live attenuated vaccine”.

Using VO, we obtained more genes and gene interactions from the vaccine-mediated IFN-γ-gene interaction network, and were also able to classify identified genes and gene interactions using the asserted and inferred hierarchies of different vaccines [11]. Another study from our group demonstrated that VO-based literature mining provided a better performance in retrieving *Brucella* vaccine-related literature and building gene interaction networks than the Medical Subject Headings (MeSH)-based approach [10]. These studies helped to generate new candidate genes for vaccine development.

The general literature mining strategy that integrates centrality and ontology has been named by us as the CONDL, standing for Centrality and Ontology-based Network Discovery using Literature data [11]. Here we report the application of the CONDL approach to retrieve gene-gene and gene-vaccine interaction networks associated with fever or vaccine-associated fever processes. Central genes and enriched biological functions are identified in these interaction networks.

## Methods

### Literature corpus

Fever-related literature published before 2011/12/31 was defined by a PubMed (http://www.ncbi.nlm.nih.gov/pubmed) query, “Fever OR Hyperthermia OR Pyrexia OR Febrile OR Pyrexial AND 1:2011/12/31[PDAT]”. The sentences in the titles and abstracts of this fever literature cohort were obtained from the BioNLP database in the National Center for Integrative Biomedical Informatics (NCIBI; http://ncibi.org/).

### Vaccine Ontology

To promote vaccine data standardization, integration, and computer-assisted reasoning, we have developed the community-based Vaccine Ontology (VO; http://www.violinet.org/vaccineontology) [11,12]. VO uses the Basic Formal Ontology (BFO) [13] as the top ontology and aligns with the Relation Ontology (RO) [14]. The VO development follows the OBO Foundry principles such as openness and collaboration [15]. The current version of VO includes over 1,000 vaccine terms against various infectious pathogens, autoimmune diseases, and cancers from over 20 human and animal species. For many of these vaccines, the vaccine components and host responses are logically defined in VO. In the current study, we used a set of 186 VO vaccine terms that are specific vaccine names at the bottom-level of the ontology hierarchy under the term “vaccine”.

### Gene and vaccine name identification

SciMiner, a dictionary- and rule-based literature mining tool (http://jdrf.neurology.med.umich.edu/SciMiner/) [16], was used to identify gene names in the fever-related literature. Identified genes were reported in terms of the official human genes based on the HUGO Gene Nomenclature Committee (HGNC) database (http://www.genenames.org/). SciMiner demonstrated 87.1% recall, 71.3% precision, and 75.8% F-measure in an evaluation using the BioCreAtIvE (Critical Assessment of Information Extraction systems in Biology) version 2 (Year 2006) Gene Normalization Task [16]. To identify vaccine names, VO-SciMiner (http://www.violinet.org/vo-sciminer/index.php), a modified version of SciMiner optimized for VO identification, was employed. VO-SciMiner demonstrated high performance in identifying VO terms; 91% recall, 99% precision, and 95% F-measure from an evaluation using 100 hand-curated gold-standard biomedical *Brucella* vaccine papers [10].

### Generation of gene-gene and gene-vaccine interaction networks

To include only gene-gene and gene-vaccine pairs with potentially true interactions rather than a simple co-occurrence in a sentence, we employed a natural language processing and machine-learning-based scoring system [11,17]. First, the sentences that contain an interaction keyword and at least two different gene names, or a gene name and a vaccine name were selected as potential interaction-describing sentences. More than 800 manually collected interaction keywords (e.g. bind, activate, interaction) were used in this study (the interaction keywords are available at: http://www.violinet.org/ifngvonet/interaction_keywords.txt). These keywords describe different types of associations between gene-gene or gene-vaccine pairs that can range from direct physical interactions (e.g., binding, ligation, attachment) to regulation relationships (e.g., up-regulation, down-regulation, and inhibition). These keywords have also been re-organized and included in an ontology structure, Interaction Network Ontology (INO) (http://purl.bioontology.org/ontology/INO).

Next, the dependency parse trees of the sentences were obtained using the Stanford Parser (http://nlp.stanford.edu/software/lex-parser.shtml) [18]. The shortest dependency path between each pair of genes (or a gene and a vaccine) in a sentence was then extracted. Support Vector Machines (SVM) [19] with an edit distance-based kernel function among these dependency paths [17] was used to classify whether a path describes an interaction between a gene or a gene-vaccine pair. Only the pairs with positive confidence scores were used to build the fever-related interaction networks [11]. In the gene-gene network, a node represents a gene. In the gene-vaccine network, a node represents either a gene or a vaccine. The nodes in these networks are connected by edges, which represent literature-derived interactions with positive SVM scores.

### Centrality analysis of networks

Centrality of a node in a network denotes how important it is in that network. The importance of a node can be defined in different ways [20]. In this study, based on the generated interaction networks, four different types of centralities were calculated: degree, eigenvector, closeness, and betweenness. Each centrality measures a specific type of importance. In degree centrality, a node is considered important if it is connected to many other nodes in the network. In contrast to degree centrality, in eigenvector centrality each neighbor does not contribute equally to the centrality of a node. A node is considered more important if it is connected to many “central” nodes. In other words, besides the quantity of the connections of a node, their quality is also taken into account. In closeness centrality, a node is more important if its total distance to the other nodes in the network is smaller. In betweenness centrality, the importance of a node is higher if it occurs on many shortest paths between other nodes. Each centrality measures a specific role of a node in a network.

Top 10 genes with highest centrality scores in each centrality measure were collected from the generic fever network and the vaccine/VO-specific fever sub-network. These most central genes from the two networks were compared against to identify those genes that are highly ranked in both networks or only in one network. These top genes were then confirmed by manually reviewing the related-literature.

### Gene set enrichment analysis

Gene set enrichment analyses (GSEA) are widely used for biological characterization of gene sets derived from high-throughput experiments including, but not limited to, microarray and RNA-seq expression studies. This is done by identifying known biological functions or functional categories such as canonical pathways or Gene Ontology terms that are over-represented in the gene set. The assessment is done using a statistical test, mostly a modified Fisher’s exact test [21] or rank-based enrichment testing [22]. The Database for Annotation, Visualization and Integrated Discovery (DAVID; http://david.abcc.ncifcrf.gov/) [23,24], a web-based functional enrichment tool, was employed in our study to identify over-represented biological functions and pathways between the genes in the fever- and vaccine-associated fever networks in terms of Gene Ontology (GO) terms and Kyoto Encyclopedia of Genes and Genomes (KEGG) pathways. Benjamini-Hochberg corrected p-value < 0.05 was used to determine a significantly over-represented term.

### Comparison of the fever gene-gene network against protein-protein interaction network

Our literature-derived fever-associated gene-gene network was compared against a network derived from other types of interactions. The data from the Human Protein Reference Database (HPRD; http://www.hprd.org) were obtained to create a protein-protein interaction (PPI) network of the literature-derived fever-associated genes from our study. The edges (interactions among the genes) were compared between the two networks. The PPI network from HPRD was incorporated into the literature-derived fever-associated gene-gene-vaccine interaction network, which enabled the generation of a new hypothesis about in-direct interactions of genes that were not directly associated.

## Results

### Study design

**Figure 1:**
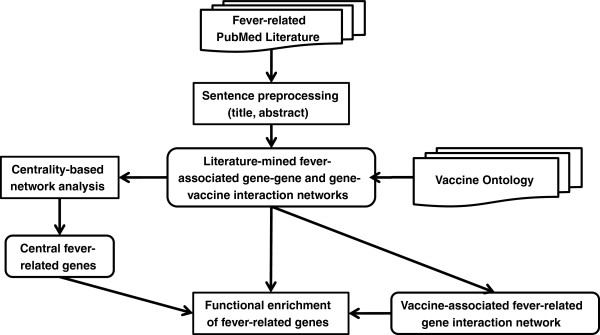
**Workflow.** CONDL workflow for discovering gene-gene and gene-vaccine interaction networks associated with fever and vaccine-associated fever events.

This study aimed to identify a generic fever gene interaction network and vaccine-related sub-networks through the centrality and ontology-based network discovery using literature data (CONDL) (Figure 1). Compared to the application of the CONDL strategy in our IFNG and vaccine-associated network extraction and analysis [11], the technical novelty of this study is that it focuses on analysis of two non-gene domains (i.e., vaccine and fever). All fever-related publications from PubMed were downloaded, and the sentences from titles and abstracts were parsed and pre-processed. Human gene names and VO terms were tagged by SciMiner and VO-SciMiner. A SVM-based method was used to identify possible interactions between gene pairs and gene-vaccine pairs. Centrality score of each gene in the interaction networks was calculated and compared among the fever-network and vaccine-associated fever-network.

### Fever-related literature-derived network

**Figure 2:**
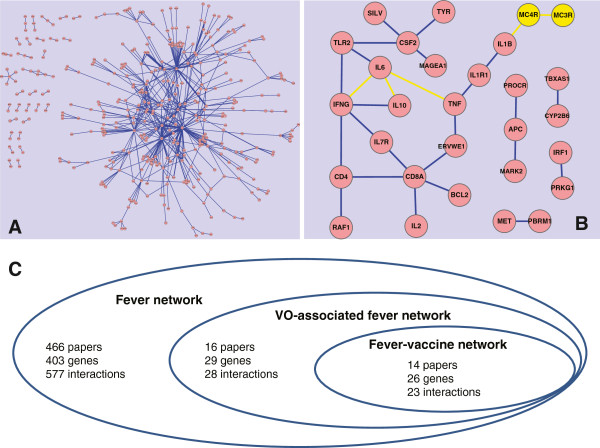
**Gene-gene interaction networks in fever and vaccine/VO-associated fever literature.** (**A**) The generic fever-related network. Thickness of edge corresponds to the number of sentences containing its respective interactions. (**B**) The vaccine/VO associated network. Nodes and edges in yellow were additionally identified by using VO terms. (**C**) Summary of the three generated gene interaction networks.

The PubMed query of fever-related literature resulted in a total of 179,156 articles (as of 12/31/2011). Two subsets of vaccine-related literature were defined with additional criteria: (1) including the terms “vaccine”, “vaccination”, and their variants (e.g., “vaccines”) (6,224 articles) or (2) including 186 specific vaccine names from VO (6,537 articles). SciMiner and VO-SciMiner were used to identify gene mentionings (symbols and names) and VO terms in the sentences of abstracts and titles of retrieved articles. Gene-gene interaction pairs at the sentence level only with a positive SVM score were used to generate three interaction networks, including the generic fever network (Figure 2A and 2C), and two sub-networks associated with “vaccine” and its derived terms per se or with expanded VO vaccine names (Figure 2B and 2C). The application of VO terms increased the numbers of retrieved papers, genes, and interactions (Figure 2C).

### Centrality analysis

All the genes in the generic fever network and vaccine/VO-specific fever sub-network were sorted based on centrality scores. The most central genes (the genes ranked among the top 10 by at least one of the centrality measures) are listed in Table 1. These genes are predicted to be associated with fever and potentially related to vaccines as well. These top genes can be grouped into three sub-sets:

(1) *Genes ranked high in both networks*. Five genes (IL1B, TNF, IL6, IFNG, and CD8A) were ranked among the top 10 in both networks by at least one centrality measure. These genes are well studied in both generic fever research and vaccine specific research [25-29].

(2) *Genes ranked high in generic fever network but not in the vaccine/VO-specific sub-network*. This group includes nine genes (HSPA1A, NFKB1, IL8, IL2, MEFV, MAPK1, POMC, CD4, and IL10). These genes have not been well studied in the vaccine context. However, since these genes are associated with fever [30-32], it can be hypothesized that many of these genes are also important in vaccine-induced fever immune responses.

(3) *Genes ranked high in vaccine/VO-specific sub-network but not in the generic fever network*. Seven genes (CSF2, IL7R, ERVWE1, APC, MC4R, IL1R1, and TLR2) were ranked high among the top 10 only in the vaccine/VO-specific sub-network. These genes have been well studied in the vaccine domain [33,34]. However, they have not been emphasized in the general fever research field. It is reasonable to hypothesize that some of the genes are also critical to other fever-associated research domains.

**Table 1:** Centrality score rankings of genes related to fever and vaccine networks

**Genes**	**Fever-network**				**Vaccine/VO-associated fever-network**			
	**D**	**E**	**B**	**C**	**D**	**E**	**B**	**C**
IL1B	1	1	1	1	---	10	8	---
TNF	2	2	2	2	7	7	1	3
HSPA1A	3	3	3	6	---	---	---	---
IL6	4	4	7	3	4	4	4	2
IFNG	5	5	4	5	1	3	3	1
NFKB1	6	6	6	4	---	---	---	---
IL8	7	9	---	8	---	---	---	---
CD8A	8	7	9	---	2	1	7	7
IL2	9	8	5	7	---	---	---	---
MEFV	10	---	---	---	---	---	---	---
MAPK1	---	10	10	---	---	---	---	---
POMC	---	---	8	---	---	---	---	---
CD4	---	---	---	9	5	6	9	6
IL10	---	---	---	10	10	---	---	9
CSF2	---	---	---	---	3	2	5	---
IL7R	---	---	---	---	8	---	---	8
ERVWE1	---	---	---	---	9	---	10	5
APC	---	---	---	---	---	5	---	---
MC4R	---	---	---	---	---	9	---	---
IL1R1	---	---	---	---	---	---	6	10
TLR2		---	---	---	6	8	2	4

The genes ranked among the top 10 by the centrality measures (*D*: Degree; *E*: Eigenvector; *B*: Betweenness; *C*: Closeness) in the fever-network and Vaccine/VO-associated fever-network. ‘---’ indicates that the gene is not ranked among the top 10 by the corresponding centrality measure in the corresponding network.

### Functional enrichment analysis

DAVID identified 997 and 239 significantly over-represented functional terms (GO or KEGG) in the fever-network and VO-associated fever-network, respectively. The top 10 most significant terms are listed in Table 2. Although the majority of the functions are significant in both sets, the fever-network was more significantly enriched with processes related to cell death (apoptosis) and responses to wounding. Considering the roles of fever, such finding is reasonable [35-41]. While “immune response” is commonly over-represented in both sets, leukocyte-related processes were the most significant in the Vaccine/VO-associated network, suggesting its important roles in vaccine-induced fever. It is interesting that the most enriched processes in the fever-vaccine sub-network are associated with positive regulation of phosphorylation and phosphate metabolic processes. How the phosphorylation process is involved in the fever and vaccines domain deserves further investigation.

**Table 2:** Top 10 most significantly enriched biological functions for each gene-gene (i.e., ‘GG’) interaction network set

**Terms**	**Fever GG network**	**Vaccine fever GG network**	**VO fever GG network**
regulation of cell death	45.5*	3.9	3.6
regulation of apoptosis	45.4*	3.9	3.6
regulation of programmed cell death	45.3*	3.9	3.6
response to organic substance	37.8*	3.4	3.9
response to wounding	36.7*	2.5	2.3
extracellular space	34.8*	1.0	1.0
positive regulation of multicellular organismal process	33.7*	3.1	3.9
defense response	33.6*	3.0	2.8
regulation of cell proliferation	31.6*	3.2	2.9
immune response	30.7*	7.8*	7.2*
cell activation	19.2	9.2*	8.7*
positive regulation of phosphorylation	16.6	7.2*	7.0*
positive regulation of phosphate metabolic process	16.3	7.2*	6.9*
positive regulation of phosphorus metabolic process	16.3	7.2*	6.9*
positive regulation of protein amino acid phosphorylation	15.3	7.3*	7.0*
leukocyte activation	14.3	7.3*	6.9*
leukocyte differentiation	9.7	8.9*	8.5*
hemopoiesis	9.6	7.3*	6.9*
lymphocyte differentiation	5.8	7.2*	6.9*

Values are –log_10_(Benjamini-Hochberg corrected P-values) from DAVID results. * corresponds to the 10 lowest p-values in each set.

### Gene-vaccine interaction networks

**Figure 3:**
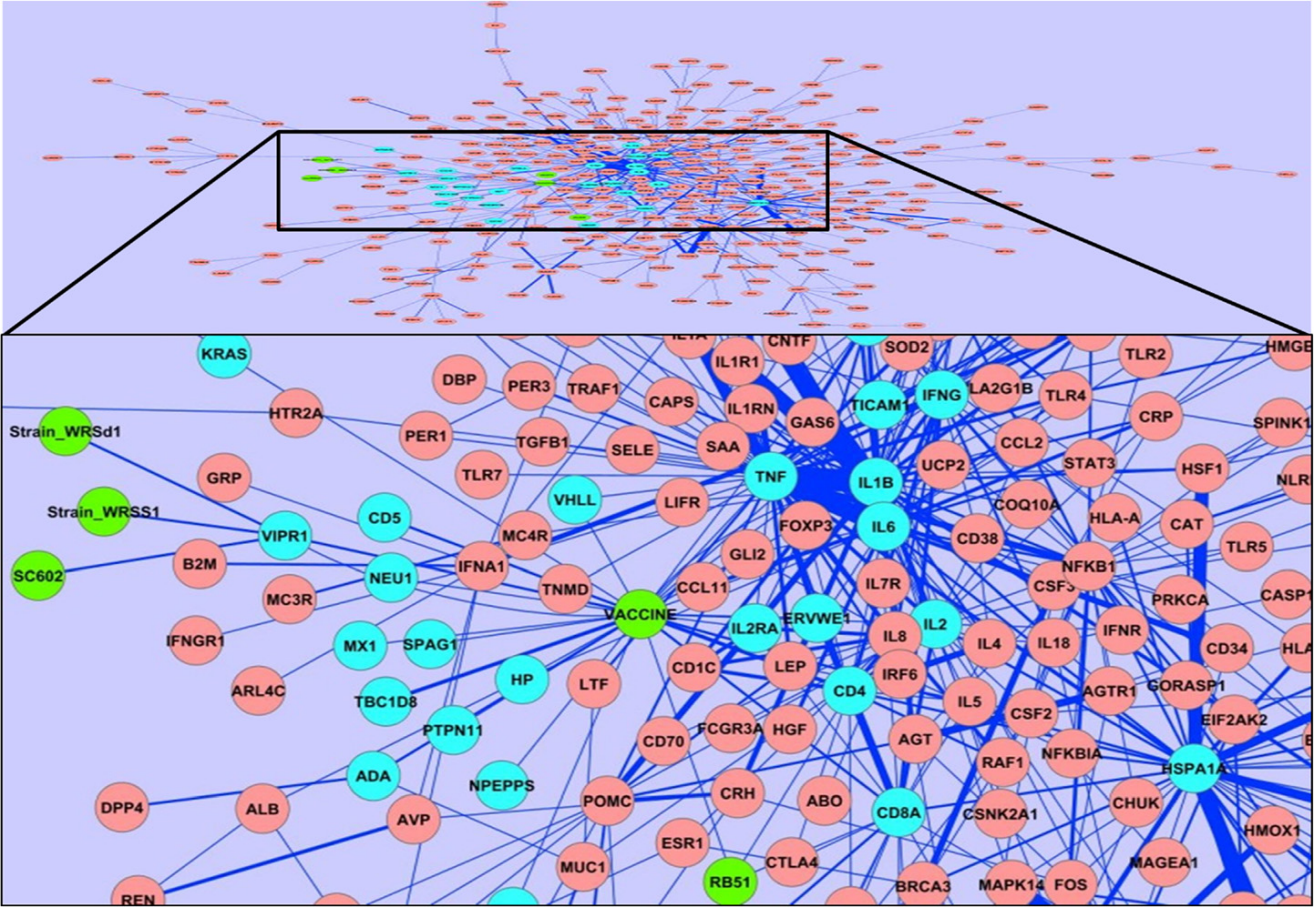
**Gene-vaccine interaction network.** Fever-related gene-vaccine interaction network incorporating all fever-related gene-gene and gene-vaccine interactions. The sub-network related to vaccine is zoomed-in. Node colors (green = vaccine; red = genes; cyan = genes associated with vaccines).

Gene-vaccine interactions with positive SVM scores were integrated into the fever networks. The whole PubMed contained 1,716 articles containing 2,835 positively-scored interactions between genes and vaccines (including VO vaccine terms). Among these articles, 32 were also related to fever, which contained 52 sentences with 44 unique gene-vaccine interactions. Specific vaccine names included *Brucella* vaccine RB51, *Shigella flexneri* vaccine SC602, *Shigella sonnei* strain WRSS1, and *Shigella dysenteriae* 1 strain WRSd1. Figure 3 illustrates the combination of gene-gene interactions and gene-vaccine interactions retrieved from the fever-associated literature (see Additional file 1 to explore the complete network).

Different levels of gene interaction networks were identified. Twenty-six genes were found to directly interact with the general term ‘vaccine’. Three of them (including CD4, CD8A, and VIPR1) also directly interact with one or more of the four vaccines. For example, *Brucella* vaccine strain RB51 induces the activation of CD4+ and CD8+ T cell responses [42]. Since both CD4 and CD8 interact with many other genes in the fever gene network, it is likely that RB51 also interacts with those genes through CD4 and/or CD8. The VO classification indicates that all these four vaccines are live attenuated vaccines. These results suggest that live attenuated vaccines are likely easier than other types of vaccines to induce fever-associated responses. These results demonstrate the other uses of VO knowledge than a simple dictionary of vaccines. These 26 genes directly interacting with vaccines can be considered as the first layer genes that interact with vaccines. The other 376 genes in the network are considered as the genes at the second layer (genes directly interacting with the 26 genes) or beyond. Although these genes have not been found to directly interact with vaccines through literature mining studies, they may indirectly interact with vaccines at different levels. Some of them may be more important than others in regulating vaccine-induced fever immune responses. It is interesting that no Toll-like receptors (TLRs) were found to directly interact with the vaccine in the fever-associated literature. Within the fever domain, many TLRs including TLR2, TLR4, TLR5, and TLR7 are found in the gene-gene interaction network. However, many TLRs are related with vaccines in the research domains not associated with fever. Therefore, it is reasonable to hypothesize that many vaccines directly or indirectly interact with these TLRs under the scope of fever domain.

### Fever-associated gene-gene network vs protein-protein interaction network

**Figure 4:**
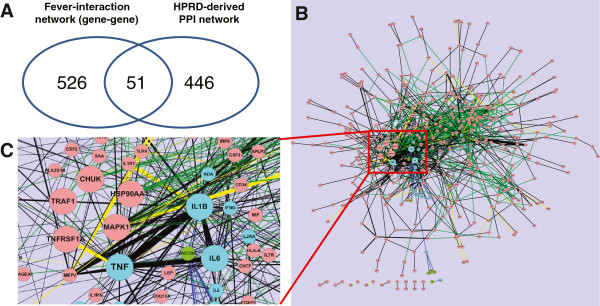
**Gene-vaccine interaction networks expanded with HPRD PPI.** (**A**) The generic gene-gene interaction network derived from fever-related literature (Figure 2A) was compared against a protein-protein interaction network generated based on the HPRD. Interactions between genes in the two networks were compared, and 51 such interactions were common to both types of networks. (**B**) The HPRD PPI network was merged into the fever-associated gene-vaccine network (Figure 3) to create a comprehensive gene-gene-vaccine interaction network in the fever and vaccine domain. (**C**) A zoomed-in network illustrates the interactions around TNF-α and TNFRSF1A, and their interacting partners. Node colors (green = vaccine; red = genes; cyan = genes associated with vaccines), edge colors (yellow = common to both networks; black = literature-derived vaccine network only; green = HPRD only).

The protein-protein interaction (PPI) information was collected from HPRD for the 403 fever-related genes obtained from our literature-based network. Among these genes, the HPRD included 497 interactions, 51 (approximately 10%) of which were common to both the literature-based and the HPRD-based PPI networks (Figure 4A).

Figure 4B illustrates an expanded fever-associated gene-gene interaction network including the protein-protein interaction data obtained from HPRD (see Additional file 2 to explore the complete network). Using the shared gene interactions in these two networks as linking points, it is possible to generate new hypotheses. For example, the interaction between TNF-α and TNFRSF1A was identified by our literature mined results and HPRD. Our literature mining approach uniquely identified the interactions of TNF-α with IL-6 and IL-1β under the vaccine domain (Figure 4C). HPRD uniquely includes the interactions of TNFRSF1A with four proteins: MAPK1, TRAF1, CHUK, and HSP90AA1. Through the connection between TNF-α and TNFRSF1A, we can hypothesize that the last four proteins (e.g., MAPK1) may also interact with IL-6 and IL-1β indirectly in the vaccine domain.

## Discussion

Vaccine-induced fever may be a positive host response to the induction of protective immunity. However, vaccine-induced fever adverse event is annoying and can even result in severe outcomes. Recently, a text mining system based on semantic tagging and rule-based techniques has been used to extract clinical features from vaccine safety reports [43]. This system has specifically been designed to extract clinical features such as diagnosis, drugs, and vaccines from the vaccine adverse event reporting system narratives. On the other hand, our current study uses the research articles in PubMed as a source and aims to identify potential gene interaction mechanisms that may contribute to vaccine-induced immune responses, including fever adverse event.

The detailed interaction network mechanisms among genes and vaccines leading to fever are not fully understood. In this study, an ontology-based literature mining approach was applied to discover gene-gene and gene-vaccine interaction networks associated with fever and fever–vaccine. Centrality and functional enrichment analyses were further employed to identify the most central genes and enriched biological functions in the networks. Our study demonstrates that ontology-based literature mining is able to efficiently discover gene-gene and gene-vaccine interactions in the fever domain.

Compared to our previous studies that analyzed the IFNγ and IFNγ-vaccine gene interaction networks [9,11], the current study focuses on analysis of fever and fever-vaccine gene interaction networks. Both studies demonstrated that the application of the VO enhanced the performance of mining vaccine-specific gene interaction networks. Therefore, the genome-wide, ontology-based literature mining approach can be applied to different domains, which can be defined by a gene name(s) (e.g., IFNγ) or by a research domain (e.g., fever).

It is noted that not many gene-vaccine interactions were obtained through our literature mining analysis. This could be due to a few reasons. Our current approach uses sentence-level SVM-positively-scored interactions. The number of hits (gene-gene or gene-vaccine pairs) will increase if we employ a simple sentence-level co-citation rather than SVM-based approach. Even more hits would be identified with an abstract level co-citation for detecting possible gene-vaccine interactions. However, these methods may result in lower specificity of detecting true interactions. The same argument applies to the identification of gene-gene interactions from the literature.

Many scientific findings and hypotheses have been generated in our study. For example, our finding of the phosphorylation-focused regulation enriched in the fever-vaccine sub-network suggests the key role of the phosphorylation process in the vaccine-induced fever phenomenon. The generic fever network has an enriched gene set in cell death regulation, while leukocyte-associated genes are overexpressed in the fever-vaccine sub-network. This suggests that leukocyte cell death is critical to vaccine-induced immunity. It is also interesting that TLRs have been studied frequently in the vaccine domain, but none of them are directly associated with fever in the literature (at least in the sentence level). Since the TLRs are included in the generic fever gene interaction network, it can be hypothesized that TLRs may be potential key factors in vaccine-induced fever responses, including fever adverse events. Therefore, our ontology-based literature mining is able to advance scientific discovery by generating new findings and hypotheses.

It should be noted that the interactions in our current approach include not only the physical interactions like PPI, but also any types of regulation and associations between genes or proteins. This is probably the primary reason why the majority of the literature-derived interactions are not identified in the HPRD PPI database. On the other hand, most of the known protein-protein interactions in HPRD are not found in our gene-gene interaction network, either. It is probably because our gene-gene interaction network is limited to the domain of fever-research, while HPRD is not. The HPRD PPI network includes any physical interactions that may not be associated with fever, or not reported in the fever-related literature.

Future research efforts will be focused on expanding our initial networks by including more specific vaccines. VO is dynamically updated and growing with the support of VO community. Increased number of VO terms will substantially improve the sensitivity of identifying gene-vaccine interactions. The method described in this paper is generic and can be applied to study other gene interaction networks in different domains. We are currently investigating the integration of our methods with the Ontology of Adverse Events (OAE; http://www.oae-ontology.org) [44,45], especially considering that the OAE development also follows the same OBO Foundry principle and design patterns as VO. We will also expand our system by integrating other adverse event-related ontologies such as OntoADR [46] and OntoEIM [47] for better understanding of the interactions between various types of medical interventions and adverse effects.

## Conclusions

Our ontology and centrality-based literature mining strategy identified genes and their potential interactions in the general fever interaction network and a subset of genes and gene interactions in the vaccine-specific fever interaction sub-network. New scientific discoveries and hypotheses were generated. This study demonstrated that ontology-based literature mining is a powerful method for studies of gene interaction networks in a specific research domain.

## Competing interests

The authors declare that they have no competing interests.

## Authors’ contributions

JH: Project design, software programming, data analysis, and drafting of manuscript; AO: Project design, software programming, data analysis, and drafting of manuscript; ZX: VO data generation, software programming, discussion, and manuscript editing. YH: Project design, data interpretation, and drafting of manuscript. All authors read and approved the final manuscript.

## Supplementary Material

Additional file 1 **Cytoscape network file for gene-vaccine interaction network.** This file contains the complete network of the literature-derived gene-vaccine interaction network. This file can be viewed using Cytoscape, an open source platform for complex network analysis and visualization, freely available at http://www.cytoscape.org/. Click here for file

Additional file 2 **Cytoscape network file for gene-vaccine interaction network with HPRD-derived PPI network integrated.** This file contains the complete network of the literature-derived gene-vaccine interaction network with the HPRD-derived PPI network integrated. This file can be viewed using Cytoscape, an open source platform for complex network analysis and visualization, freely available at http://www.cytoscape.org/. Click here for file
